# DEMENTIA FAMILY CAREGIVERS’ EXPERIENCES WITH A NURSE-LED MEMORY CARE CLINIC

**DOI:** 10.1093/geroni/igz038.408

**Published:** 2019-11-08

**Authors:** Mariya A Kovaleva, Bonnie M Jennings, Carolyn Clevenger, Mi-Kyung Song, Patricia C Griffiths, Ken Hepburn

**Affiliations:** 1 Vanderbilt University School of Nursing, Nashville, Tennessee, United States; 2 Nell Hodgson Woodruff School of Nursing, Emory University, Atlanta, Georgia, United States; 3 Emory University, Atlanta, Georgia, United States; 4 Emory University School of Medicine, Atlanta, Georgia, United States

## Abstract

The Integrated Memory Care Clinic (IMCC) at Emory Healthcare is a patient-centered medical home led by advanced practice registered nurses (APRNs) who provide both dementia care and primary care. We explored the experiences of informal caregivers of persons living with dementia at the IMCC during their first year post-enrollment. Twelve caregivers completed semi-structured telephone interviews that lasted 29 minutes on average. The data were analyzed via directed content analysis guided by attention only to caregivers’ accounts of their experience at the IMCC. Caregivers’ experiences clustered around two major considerations: the strengths of the IMCC, and ways to enhance the IMCC. Overall, caregivers’ viewed the IMCC as their wished-for care model. Caregivers felt a sense of belonging to the IMCC team, as they understood that the IMCC personnel incorporate caregivers’ input to deliver care. Participants valued APRNs’ competence in dementia care and having direct telephone access to an on-duty APRN around the clock. Caregivers appreciated the care organization at the IMCC with adequate time dedicated for in-person visits. Areas for the IMCC improvement included clarifying the IMCC scope of practice, explaining dementia progression, involving physicians, and providing more medical and non-medical resources at the IMCC. Caregivers’ willingness to have more resources provided by the IMCC emphasizes how many unmet needs caregivers and their persons have. Clarification of the clinics’ scope of practice – what can be done to manage dementia, its symptoms, and comorbidities – highlighted the need to educate caregivers about ways in which dementia, albeit incurable, can be managed.

